# Gestures convey different physiological responses when performed toward and away from the body

**DOI:** 10.1038/s41598-019-49318-3

**Published:** 2019-09-06

**Authors:** Angela Bartolo, Caroline Claisse, Fabrizia Gallo, Laurent Ott, Adriana Sampaio, Jean-Louis Nandrino

**Affiliations:** 1 Univ. Lille, CNRS, CHU Lille, UMR 9193 - SCALab - Sciences Cognitives et Sciences Affective, F-59000, Lille, France; 2 Institut Universitaire de France, Paris, France; 3 Psychological Neuroscience Lab, CIPsi, School of Psychology, University of Minho, Campus de Gualtar, 4710-057 Braga, Portugal

**Keywords:** Neuroscience, Human behaviour

## Abstract

We assessed the sympathetic and parasympathetic activation associated to the observation of Pantomime (i.e. the mime of the use of a tool) and Intransitive gestures (i.e. expressive) performed toward (e.g. a comb and “thinking”) and away from the body (e.g. key and “come here”) in a group of healthy participants while both pupil dilation (N = 31) and heart rate variability (N = 33; HF-HRV) were recorded. Large pupil dilation was observed in both Pantomime and Intransitive gestures toward the body; whereas an increase of the vagal suppression was observed in Intransitive gestures away from the body but not in those toward the body. Our results suggest that the space where people act when performing a gesture has an impact on the physiological responses of the observer in relation to the type of social communicative information that the gesture direction conveys, from a more intimate (toward the body) to a more interactive one (away from the body).

## Introduction

Manual gestures are used to interact with tools or to communicate. Two types of manual gestures have attracted the attention of the scientific community in the last few years, namely Intransitive and Pantomimes. Intransitive gestures are expressive or symbolic actions and are used to express a feeling (e.g. “I’m cold”) or a command together or in the absence of language (e.g. “go away”). Pantomime is the gestural description of tool use and is performed to represent the tool use in the absence of it. Both Intransitive and Pantomime gestures share a communicative intent; the first are used to describe a feeling or to give a command, whereas Pantomime gestures are performed to allow the observer/executor to recognize/perform the action. Pantomime and Intransitive gestures are therefore special entities in that on one side they are motor actions and on the other they have a communicative intent^1^. The motor side of gestures and their communicative intent are interrelated. Hand posture, configuration, orientation and movement have to be appropriately implemented to allow the observer/executor to understand/produce the Intransitive gesture or the Pantomime. For instance, if the gesture of “stop” (Intransitive gesture) is not correctly executed by extending the arm and placing the back of the hand in front of the face, other meanings (or no meaning) can be attributed to the gesture: if the person raises the hand instead of placing it in front of him/her, it can be interpreted as a gesture of “asking for attention”. Similarly, for the Pantomime of the use of a comb, the hand has to be configured in a fist, placed at a certain distance from the hair and a vertical movement along the head has to be performed. In this way, a person can recognize/perform the combing movement. If the hand remains open, the gesture might resemble to a caress. When produced in context, the gesture of combing invites the observer to comb her/his or to find a comb (Pantomime); the gesture of “coming here” invites a person to approach (Intransitive). However, the motor program to perform Pantomime and Intransitive gestures differs: to perform the Pantomime one has to bear in mind the tool features as well as its functional properties to better configure the hand and to perform the appropriate gesture; whereas Intransitive gestures are stored in long memory, are culturally dependent and can be expressed in different ways (the gesture of “that’s fine” can be performed with the gesture of “thumb up” or the gesture of “ok”).

The ability to produce or to understand gestures can be compromised as a consequence of brain damage, commonly observed as a result of a lesion in the left hemisphere. The resulting disability in gesture comprehension and production is called limb apraxia, considered a deficit in processing gestures in the absence of other cognitive disabilities that could explain the deficit (e.g. motor and sensory problems, tool recognition deficits, response implementation^2^). Neuroimaging studies showed that the processing of both gesture types activates parietal regions^3–5^, yet neural dissociations have been reported: premotor and occipito-parietal damages are more sensitive to deficits in Pantomime^3,4^ whereas anterior temporal lesions affect Intransitive gestures^4^.

In terms of cognitive demands, some studies identified the role of working memory skills^6^ or motor imagery^7^ in Pantomimes, whereas Intransitive gestures are supposed to rely on social cognitive abilities^8,9^. Halsband and collaborators^10^ found that patients with left parietal lesion had more difficulty to perform Intransitive and Pantomime gestures toward the body than those performed away from the body. The authors considered this result coherent with previous findings^11^ showing that apraxic patients display deficits in the coding and representation of movements in relation to their own body-scheme, whereas the action in the extrapersonal space are better preserved^12^. The space surrounding our body is indeed not homogeneous but distinguished into functionally distinct neural representations^13,14^. In particular, the peripersonal space is the space immediately surrounding our body and objects located in this space can be reached and grasped^15^. Crucially, an object can be grasped to be manipulated away from the body (e.g. a pair of scissors) or to be used toward the “bodily space” (e.g. toothbrush); therefore, the (functional) meaning of the stimulus defines in which peripersonal space it will be mapped, that is in our “protective space” (body reference) or in our “working space” (specific of goal-directed actions^14^, p. 327).

Concerning Intransitive gestures, Gallagher and Frith^16^ distinguished instrumental and inner gestures. The formers are used to change the behavior of others by communicating commands and it is to note that they are usually performed away from the body (e.g. “come here”; “go away”). Inner gestures represent feeling states and they are often gestures performed toward the body (e.g. “I feel cold”, “I feel hot”). Using fMRI, they found that inner gestures elicited activity in a neural network considered part of the social brain, including the bilateral amygdala. In contrast, instrumental gestures elicited activity in a left-lateralized system previously associated with language processing (inferior frontal gyrus)^16^. Therefore, since inner gestures inform the observer about the feeling of the executor, we might state that these gestures allow them to pierce the intimate state of the person producing the gesture. On the other hand, instrumental gestures rely on a non verbal communication system and are used to directly interact with the individuals. Rephrasing the above statement on space representation, the meaning of the Intransitive gesture might vary according to where the gesture is directed.

Concerning Pantomimes, when such gestures are performed toward the body they are usually associated to personal hygiene or nutrition (brushing teeth, putting on makeup; combing hairs; drinking etc.). Therefore, similar to Intransitive gestures, Pantomimes performed toward the body disclose the intimate/private sphere of the individual that performs the action. Pantomimes executed away from the body reveal an action that might be performed with another object (e.g. hammering with a nail) and the executor has to make understandable to the observer the tool s/he is using in the absence of it. The information they carry is restricted to the functional meaning of the action and the observer is engaged in a sort of detection of the tools used in the “working space” of the individual.

In summary, both Intransitive and Pantomime gestures should be differently perceived when performed toward and away from the body in agreement with the different meaning they convey.

A recent meta-analysis study showed that Intransitive gestures performed during face to face interaction involve brain regions associated to social emotional processes (inferior frontal gyrus, putamen and insula)^17,18^. This finding confirms that gestures express the producer’s feeling. Since gestures convey social-emotional information, psychophysiological measures may represent an appropriate tool to investigate the physiological modifications that occur during gesture observation.

To investigate how the perception of gesture toward and away from the body varies, two physiological measures have been employed, pupillometry and heart rate variability (HRV).

Pupillometry is used to detect pupil dilatation (or constriction) and is a physiological index of the autonomic activity that is critical at early stages of emotional responses^12,19,20^. Variations of pupil size are under the control of two smooth muscles, the sphincter papillae for its constriction and the dilator papillae for the dilation. Pupil dilation is provoked by the activity of the sympathetic system that stimulates the dilator papillae muscle and or by a simultaneous parasympathetic activity that inhibits the sphincter papillae muscle^21,22^. Pupil dilation has been observed in infants in relation to irrational social actions^23,24^; it has been used to reveal empathy capacity in 2-year-olds^25^, suggested to reflect emotional arousal^26^ for both pleasant and unpleasant stimuli^19^. HRV corresponds to the dynamical changes in the time periods between adjacent heartbeats and is used to investigate cardiac vagal tone. It represents the contribution of the sympathetic and parasympathetic nervous system to cardiac regulation. It reflects the physiological ability to adapt to the modification of the environment and it is largely used in studies with socio-emotional stimuli^27,28^ including self-regulation at the cognitive, emotional, social and health levels. More specifically, in the framework of the frequency-domain analysis, high frequencies (HF-HRV) between 0.15 and 0.40 Hz represent a valid and reliable marker of the parasympathetic activity and of the vagal tone^29,30^ and would reflect the level of cognitive, behavioral and emotional regulatory abilities^31,32^. An adequate cardiac control reveals a flexible engagement with the environment^33,34^. Furthermore, heart rate variability has been also used as a biomarker of self regulation and cognitive control in psychopathology^35^.

In this study, participants have been asked to passively observe a series of gestures (Intransitive, Pantomime) performed toward and away from the body while pupil dilation and HF-HRV were recorded. Meaningless gestures have been also included. These gestures are unfamiliar and carry no meaning (“put the hand under the chin”) and since they do not convey any social-emotional communicative information, they are included as control gestures and no physiological distinction between gestures performed toward and away from the body is expected.

Results on HF-HRV and pupil dilation should bring to light a difference in gesture meaning when the direction of the gesture is taken into account. More specifically, as pupil dilation is more sensible to detect social-emotional states (i.e. emotion and empathy); we predict large pupil size for meaningful gestures (i.e. Intransitive and Pantomime) performed toward the body, having the status of providing information on the personal/intimate sphere of an individual. Intransitive gestures executed away from the body should elicit a motor response/reaction of the observer that should affect his/her homeostasis; therefore HF-HRV should be higher for Intransitive gestures executed away from the body with respect to the same gestures performed toward to the body. Since Pantomime away from the body are supposed not to modify the behavior of others, no difference between the two directions for this gesture type is expected.

## Results

### Pupil dilation

**Figure 1:**
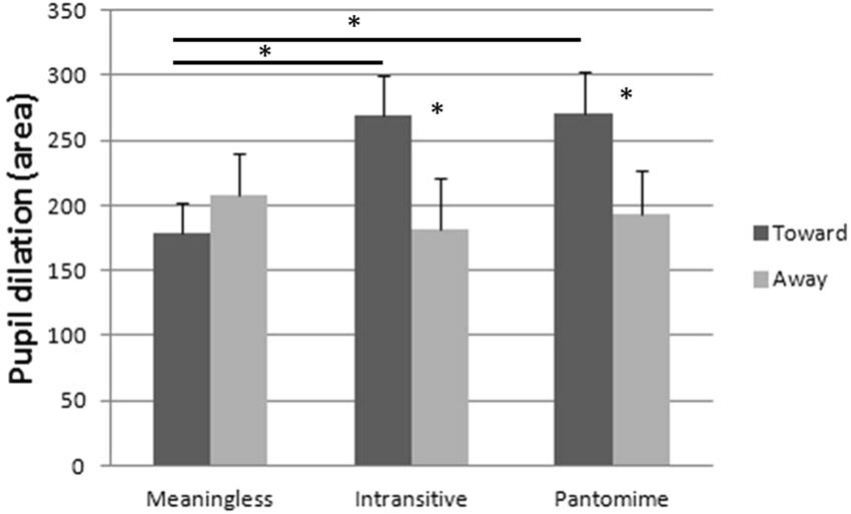
Size of pupil dilation in the three gesture conditions (Meaningless, Intransitive and Pantomime) according to their direction (Toward and Away from the body). Vertical bars represent the SEM. Asterisks indicate significant differences.

Data on pupil dilation were normally or were close to normally distributed (Shapiro-Wilk: Meaningless toward, *W* = 0.93, *p* = 0.04, and away from the body, *W* = 0.99, *p* = 0.95; Intransitive toward, *W* = 0.97, *p* = 0.59, and away from the body, *W* = 0.97, *p* = 0.64; Pantomime toward *W* = 0.93, *p* = 0.04, and away from the body, *W* = 0.95, *p* = 0.14). Since results did not change when using non parametric analyses (namely Friedmann ANOVA and Wilcoxon t-test), a 3 (Gesture type: Meaningless, Intransitive and Pantomime) × 2 (Direction: toward and away from the body) ANOVA for repeated measure was run. Mauchly’s test indicated that the assumption of sphericity was not violated (Gesture type: *χ*^2^(2) = 0.67, *p* = 0.71; Gesture type × Direction: *χ*^2^(2) = 4.41, *p* = 0.11). The main effect of Gesture type was not significant, *F*(2, 60) = 1.26, *p* = 0.29, η_p_^2^ = 0.04; yet the main effect of Direction was, *F*(1, 30) = 5.94, *p* = 0.021, η_p_^2^ = 0.17. Pupil dilation was higher for gestures toward (*M* = 239.2 *SD* = 134.0) than away the body (*M* = 194.2 *SD* = 145.6). The interaction Gesture type × Direction was also significant, *F*(2, 60) = 3.81, *p* = 0.03, η_p_^2^ = 0.11. Fisher post hoc analyses showed that Meaningless gestures toward the body (*M* = 178.2 *SD* = 135.2) did not differ from Meaningless gestures away from the body (*M* = 208.3 *SD* = 179.4) but they differed from both Intransitive (*M* = 269.3 *SD* = 170.5; *p* = 0.008) and Pantomime gestures toward the body (*M* = 270.1 *SD* = 181.3; *p* = 0.008). The Intransitive gestures toward the body differed from both Intransitive (*M* = 181.2 *SD* = 218.9; *p* = 0.01) and Pantomime gestures away from the body (*M* = 193.2 *SD* = 183.4; *p* = 0.03); and Pantomime gestures toward the body differed from Pantomime (*p* = 0.02) and Intransitive gestures away from the body (*p* = 0.01) (*cf*. Fig. 1).

### Hearth rate variability (HF-HRV)

**Figure 2:**
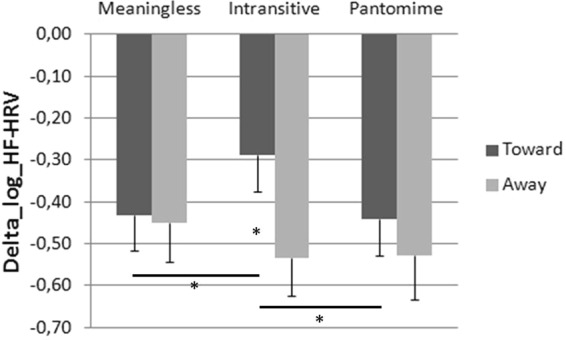
ΔHF-HRV in the three Gesture conditions (Meaningless, Intransitive and Pantomime) according to their direction (Toward and Away from the body). Vertical bars represent the SEM. Asterisks indicate significant differences.

Data (Δ HF-HRV, with Δ = HF-HRV – Baseline) distributed normally (Shapiro-Wilk: Meaningless toward, *W* = 0.95, *p* = 0.15, and away from the body, *W* = 0.97, *p* = 0.52; Intransitive toward, *W* = 0.96, *p* = 0.22, and away from the body, *W* = 0.97, *p* = 0.45; Pantomime toward *W* = 0.97, *p* = 0.63, and away from the body, *W* = 0.96, *p* = 0.33) and were analyzed using a 3 (Gesture type: Meaningless, Intransitive and Pantomime) × 2 Direction (toward and away from the body) ANOVA for repeated measure. Mauchly’s test indicated that the assumption of sphericity was not violated (Gesture type: *χ*^2^(2) = 0.46, *p* = 0.79; Gesture type × Direction: *χ*^2^(2) = 1.28, *p* = 0.53). Results did not reveal any effect of Gesture type, *F*(2, 64) = 0.97, *p* = 0.38 η_p_^2^ = 0.03, but the main effect of Direction was significant, *F*(1, 32) = 14.07, *p* < 0.001 η_p_^2^ = 0.31, with larger HR variability for gestures away from the body (*M* = −0.50, *SD* = 0.50) with respect to those toward the body (*M* = −0.39, *SD* = 0.44). The interaction Gesture type X Direction was also significant *F*(2, 64) = 3.50, *p* = 0.036 partial η_p_^2^ = 0.10, *cf*. Fig. 2). Fisher post hoc analysis showed that Intransitive gestures toward the body (M = −0.29, SD = 0.49) differed from all the other gesture types:

- Intransitive gestures away from the body (*M* = −0.53, *SD* = 0.53; *p* = 0.0002);
- Meaningless gestures toward (*M* = −0.43, *SD* = 0.47; *p* = 0.02) and away from the body (*M* = −0.45, *SD* = 0.53; *p* = 0.01);
- Pantomime gestures toward (*M* = −0.44, *SD* = 0.50; *p* = 0.02) and away from the body (*M* = −0.53, *SD* = 0.59; *p* = 0.0003).

## Discussion

Pupil dilation and HF-HRV have been recorded in a group of healthy participants while they passively observed a person performing Meaningless, Intransitive and Pantomime gestures toward and away from the body without producing any facial emotional expression. The aim of this study was to investigate whether the direction of the gestures (toward and away from the body) produced variations in pupil dilation and HF-HRV especially in meaningful gestures (Intransitive and Pantomime).

Results showed that pupil dilation did not vary according to the gesture type, but it modified according to its direction (η_p_^2^ = 0.17). The effect of the direction was qualified by the significant interaction Gesture × Direction (η_p_^2^ = 0.11). The interaction was driven by the meaningful gestures, as no difference between Meaningless gestures toward and away from the body was observed. As predicted, both Intransitive and Pantomime gestures toward the body showed greater pupil dilation with respect to gestures away from the body. Gestures toward the body are performed in the personal (bodily) space of the individual and reflect hygienic or nutritional activity when Pantomimes are performed (e.g. “combing oneself” or “eating a soup”) or mental states in the case of Intransitive gestures (e.g. “being hungry”; “vomiting”). In both cases, gestures belong to the private area of the individual that performs the action. A previous neuroimaging study showed that at least Intransitive inner gestures (usually performed toward the body) activate brain regions associated to theory of mind^16^ that included the bilateral amygdala. The amygdala is described to play a role in pupil dilation by stimulating the sympathetic input or by inhibiting the parasympathetic one^21,22,36,37^. Hence, since Intransitive gestures are particularly sensitive to elicit the activity of the amygdala, pupil dilation might be interpreted as a consequence of the activity of the amygdala on the sympathetic or parasympathetic system.

Similar to Intransitive gestures, the observation of Pantomimes toward the body produced comparable pupil dilation. Recent studies suggested a link between Pantomimes and social-emotional deficits. A recent one found that children with autism spectrum disorder were specifically impaired in Pantomime production and the severity in the execution of Pantomimes correlated with the severity of social deficits^38^. In another study, it has been found that patients with schizophrenia displaying non verbal social perception deficits also produced gestural deficits, including difficulties in Pantomime production^39^. Although these studies did not propose a distinction between gestures toward and away from the body, a perusal to the list of gestures used showed that both gesture directions were included.

Together, our results suggest that, since pupil dilation indexes a social-emotional process, Intransitive and Pantomimes gestures toward the body should convey social-emotional information. More specifically the observation of these gestures might reveal the private affairs of the individual performing the action.

Furthermore, we found that the direction of the gestures provoked high Delta HF-HRV for gestures away from the body (η_p_^2^ = 0.31), however the significant gesture × direction interaction (η_p_^2^ = 0.10) revealed that the effect was driven by the low level of vagal suppression (i.e. decrease of Delta HF-HRV) registered in Intransitive gestures toward the body. The level of vagal suppression in both Pantomime and Meaningless gestures was similar in both gesture directions. Only for Intransitive gestures, the activity of the parasympathetic system was modulated by gesture direction, with a high vagal suppression for gestures away from the body. Previous studies showed that the interaction with the environment produces HRV modification in response to the processing of social emotional information^40^. The significant effect of direction with a specific increase of the Delta HF-HRV in the gestures away from the body might indicate that our participants need more social emotional regulatory processes during the observation of these social gestures. Since the increase of Delta HF-HRV is modulated by gesture direction only in the Intransitive gestures, this result might be interpreted considering the meaning of these gestures. With respect to Meaningless and also Pantomimes, Intransitive gestures are specifically executed to interact. However, Intransitive gestures toward and away from the body convey different information when interacting with others. When Intransitive gestures are performed toward the body, they inform the observer of the mental state of the individual (e.g. “I’m cold”). These gestures do not require the observer to provide a motor action in response to the observed gesture, s/he can be passive while the gesture is observed and understood. For instance, if someone is performing a gesture of perplexity (“scratching the head”), the observer is not asked to intervene. Therefore, low vagal suppression is observed since no self-regulation processes are required. Unlikely, Intransitive gestures performed away from the body (e.g., “come here”, “go away” etc.) have the peculiarity to elicit a motor response of the observer, in that they are used to actively interact with the individuals that are obliged to change their behavior in response to the observed gesture. Given that, the observation of Intransitive gestures performed away from the body would elicit social emotional regulatory processes as a consequence of the social meaning of the gesture.

Nonetheless, results also show high Delta HF-HRV in Meaningless and Pantomime gestures which was similar in the two directions (toward and away from the body) but also similar to the one observed in Intransitive gestures away from the body. This result was not expected, as Pantomime and Meaningless gestures are not performed to enhance a motor response of the observer. One possibility is that the process that provokes an increase of Delta HF-HRV in Pantomime and Meaningless gestures is not the same that produces an increase of Delta HF-HRV in the Intransitive gestures away from the body. Since the increase of Delta HF-HRV corresponds to the classic vagal response in a situation of uncertainty or stress^32^, the increase of Delta HF-HRV in Pantomime and Meaningless gestures might be related to the features of these gestures that are unfamiliar and artificial, thus perceived with a high level of uncertainty. This possibility is supported by the results achieved in our pilot study in which RT were higher when the gesture was meaningless than meaningful. Differently from Pantomime and Meaningless gestures, the Intransitive ones are unambiguous and easy to perform^41^; therefore, the increase of Delta HF-HRV during the observation of Intransitive gestures away from the body fits well with the above hypothesis that the observation of these gestures entails a rapid behavioral adjustment that allows the observer to react to the gestural message. The large vagal suppression should be the result of this behavioral adjustment^42^. A way to explore the possible different meaning of the large vagal suppression in the three gesture types would be to collect some cognitive measures aimed at exploring working memory and attentional processes that are required when tasks are difficult to execute. If the increase of Delta HF-HRV in Pantomime and Meaningless gestures is related to the level of uncertainty due to the complexity of these gestures, a correlation with the scores at the working memory and attentional tasks should be found only for these two gesture types regardless of gesture direction. On the contrary, the increase of the Delta HF-HRV in Intransitive gestures away from the body should not correlate with these cognitive measures. However, in the absence of complementary information, such interpretation remains speculative and deserves to be further explored.

Taken together, our results suggest that the “bodily space” cannot be conceived only as a defensive or protective space^14^, as this space is an area when individuals can act toward themselves to deliver a private information to the observer or to perform personal activities (e.g. nutrition, hygiene).

In summary, the results of this study suggest that the psychophysiological state changes when observing Pantomime and Intransitive gestures directed toward and away from the body. In particular, the perception of both Pantomime and Intransitive gestures toward the body deliver private information concerning the individual performing the action which is qualified by an increase of pupil dilation in the observer. When Intransitive gestures are performed away from the body, social emotional regulatory processes (increase of Delta HF-HRV) are involved in the observers probably because the observation of these gestures elicits in turn a social motor response. Importantly, these results have been obtained while participants observed an actress performing gestures in a neutral way, as no facial emotional expression was produced while performing the action. This means that the meaning of the gesture is enough to elicit social-emotional activations in the observer.

As a limitation, in this study our participants visualized video-gestures performed by an actress, thus the gestures’ executor was a female person for the whole duration of the study. Furthermore, our sample was mainly composed of female participants (26 of the 33 participants). Future studies should address the question of a gender effect in gesture observation.

## Methods

### Participants

We calculated that a sample size of 29 participants would be sufficient in our study to detect a significant effect with a power of 0.90 and an alpha of 0.05 with both pupil dilation and HF-HRV measures (G*Power^43^).

Thirty-three French native healthy participants (26 females) between 18 and 25 years old entered the study (26 females, Age: *M* = 21.5, *SD* = 2.7; Handedness: Range: −0.7–1, *M* = 0.64, *SD* = 0.37; Oldfield, 1976).

For technical problems, data on pupil dilation were not available for 2 participants, therefore data analyses on pupil dilation have been run on 31 subjects (25 Females, Age: *Range* = 18–25, *M* = 21.5, *SD* = 2.7; Handedness: Range = −0.7–1, *M* = 0.65, *SD* = 0.37^44^).

Their vision was normal or corrected-to-normal.

Participants were asked to fill a questionnaire for the assessment of anxiety and depression (Hospital Anxiety and Depression Scale, HADS^45^), and two questionnaires for the evaluation of emotion regulation abilities (Difficulties in Emotion Regulation Scale, DERS-F^46^ and Cognitive Emotion Regulation Questionnaire, CERQ^47^) to rule out the presence of mood disorders. Demographic data and results on the questionnaires are reported in Table 1.

**Table 1:** Results on the emotional questionnaires.

Questionnaires (Number of participants)		Participants
		Range, Mean (*M*) and Standard Deviation (*SD*)
**CERQ (N** **=** **33)**	*Total Adaptation*	50–92, *M* = 70.8 *SD* = 11.1
	*Total Non Adaptation*	23–58, *M* = 38.4 *SD* = 8.5
**DERS (N** **=** **32)**		34–80, *M* = 49.9 *SD* = 10.6
**HADS (N** **=** **33)**	*Total Depression*	0–9, *M* = 4.12 *SD* = 2.6
	*Total Anxiety*	4–20, *M* = 9.16 *SD* = 3.6

The study was conducted in a quiet room of the Laboratory SCALab at the University of Lille (France). All the participants gave written informed consent to participate in the study.

The experimental protocol was approved by the local ethical committee in behavioral sciences of the University of Lille (Ref. number 329-S69) and conducted in accordance with the Declaration of Helsinki.

### Material

An actress was asked to perform a series of gestures (Meaningless, Intransitive and Pantomime) toward and away from the body. To prevent any confounding factors in the experimental design, she was asked to avoid facial emotional expression while performing the gestures. The initial dataset of videos included 166 gesture-videos of 4 s each. Half of the gestures (n = 83) was performed with the right hand and the other half was composed of the same gestures performed with the left hand. The background of the video was set in green (RGB 86 156 87) to enhance the contrast with the actress performing the gesture. To select the stimuli, a pilot study including 14 French naive university students (Age: *Range* = 21–27, *M* = 24.3 *SD* = 2.1) was run. Participants were asked to visualize the videos that were run with PsychoPy v1.90.2. Each gesture-stimulus was presented one after the other with 3 s of inter stimulus interval. Participants were asked to say whether the gesture was meaningful or not (“Is this gesture meaningful?”) by using the AZERTY keypad of a 15” DELL laptop. Gesture-videos were presented in a random order. Participants pressed the “Q” key for yes and the “M” for no answers; answers were counterbalanced across participants (“Q” for no and “M” for yes answers). The experiment was split into two parts to avoid fatigue and it was run in one session. Response time and accuracy were registered.

**Figure 3:**
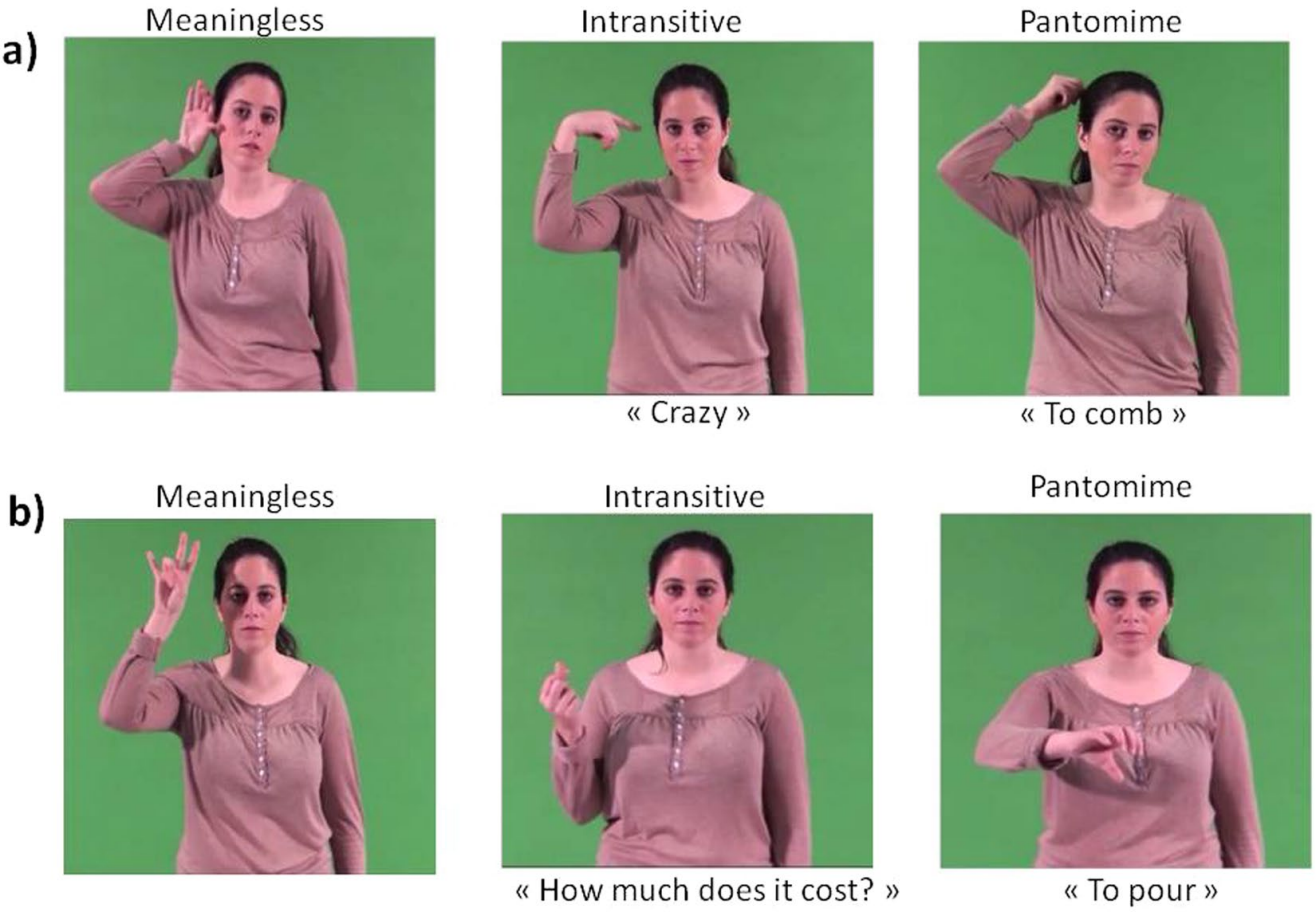
Examples of gestures used (frames from the videos): (**a**) Toward the body; (**b**) Away from the body.

Sixty gesture-videos have been selected, 20 for each gesture category (Meaningless, Intransitive and Pantomime, see Fig. 3 for some examples and the Supplementary Information for a full list of the gestures used). Half of them was performed with the right hand and the other half was the same gestures performed with the left hand. Then, half was executed toward the body, the other half away from the body. Overall, no difference between gestures performed with the right and left hand in terms of response time, *t*(13) = 1.1, *p* = 0.31 and accuracy, *t*(13) = 0.59, *p* = 0.56, was observed, therefore, response time and accuracy registered in both hands were grouped together for the following analyses. Since data on RT normally distribute (skewness range: −0.62 to 0.92; kurtosis range: −1.1 to 1.8) and sphericity assumption was not violated (Mauchley test p > 0.5), a 3 (Gesture type: Meaningless, Intransitive and Pantomime) X 2 (Direction: toward and away from the body) ANOVA for repeated measures was run on response time. Results showed only a main effect of Gesture type, *F*(2, 26) = 7.97, *p* = 0.002 *η*_*p*_^2^ = 0.38. No main effect of Direction, *F*(1, 13) = 3.1, *p* = 0.11 *η*_*p*_^2^ = 0.19 and no interaction Gesture type × Direction was observed, *F*(2, 26) = 2.1, *p* = 0.14 *η*_*p*_^2^ = 0.14. Fisher Post hoc analyses showed that participants took more time to respond to Meaningless (*M* = 2.72 s, *SD* = 0.28) than to both Intransitive (*M* = 2.45 s, *SD* = 0.27; *p* < 0.001) and Pantomime gestures (*M* = 2.57 s, *SD* = 0.29, *p* = 0.04), with no difference between Intransitive and Pantomime gestures (*p* = 0.10). It takes more time to provide “no” answers than to affirm that a meaning is present in the gesture observed. In terms of accuracy, data did not normally distribute. Friedmann ANOVA for repeated measures did not show any significant difference among the 6 Gesture types, *χ*^2^(5) = 6.1, p = 0.29 (*Mdn* = 100% for each gesture).

Additionally, 10 gesture-videos were also selected to be presented as first during the experiment, containing a mix of the three conditions (2 Meaningless away and 2 toward; 2 Intransitive toward and 1 away; 2 Pantomime toward and 1 away). Results on this “trial block” of stimuli were not considered for the analysis as it served to reduce the physiological response enhanced by the visualization of the first set of stimuli.

### Procedures

Participants entering the experiment were volunteers and were recruited at the University of Lille (France) via advertisements. They were given a letter of information defining the experimental setting and providing explanations on the experiment. Participants were then invited to show up for the experiment and asked to avoid all psychoactive substance (i.e, coffee, tea, tobacco, alcohol) one hour before. The experiment was carried out in the morning in a SCALab experimental box between 10 am and 12 am or in the afternoon between 2 pm and 4 pm in order to reduce the effects of circadian rhythm on collected measures. Room temperature in the laboratory throughout the study was held constant at 24 °C. Once signed the consent form, the experiment could start.

Physiological measures of the two branch of autonomic nervous system need different time of assessment. Concerning the emotional sympathetic response, the pupil dilation is recorded in short time window of 2 to 5 s after the stimulus onset. Concerning the parasympathetic response, HRV allows us to measure a regulatory response. This response needs more time to regulate activation; therefore each 4 s gesture-video was repeated twice.

**Figure 4:**
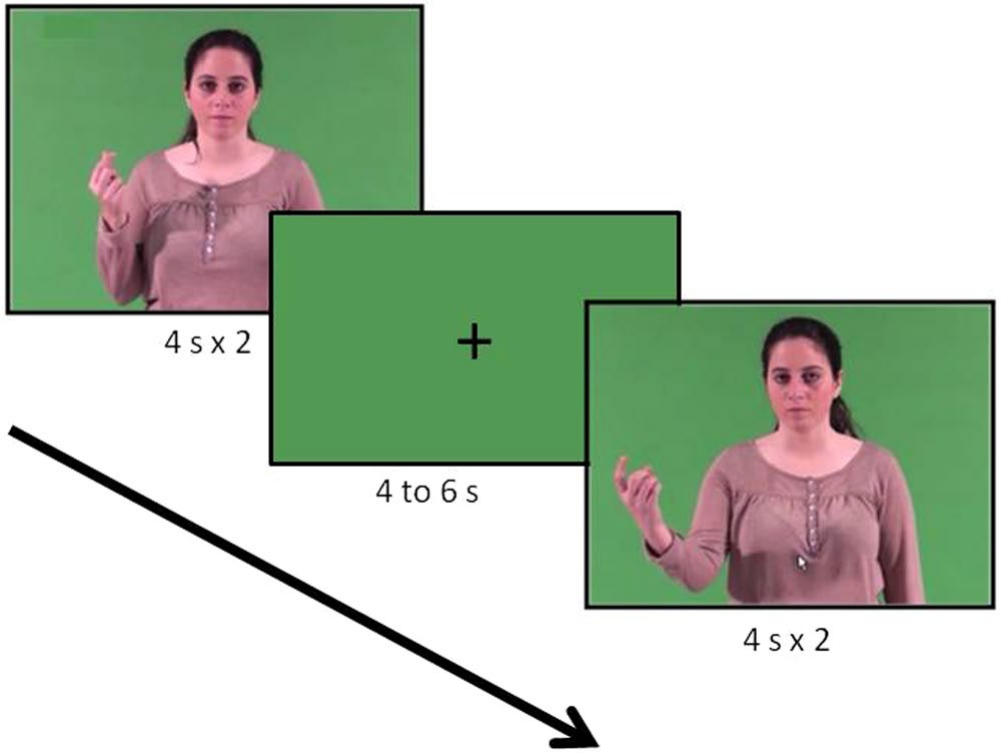
Example of trial.

The stimulus was the 4-s gesture-video repeated twice one after the other, thus the duration of the stimulus for this experimental part was 8 s. Each gesture-video was followed by a green screen of the same color as the background in the videos (RGB 86 156 87) containing a fixation cross (“ + ”) lasting 4, 5 or 6 s (pseudo-random order, Fig. 4).

**Figure 5:**
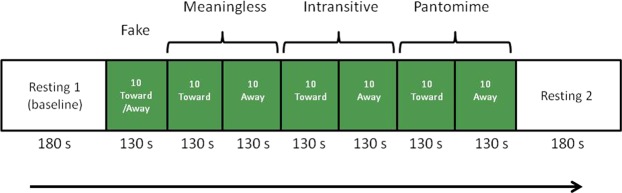
Flow of the experiment.

Each Gesture condition (Meaningless, Pantomime and Intransitive) contained two Direction blocks of 10 gesture-video, one for each gesture direction (10 toward and 10 away from the body), lasting 130 s each (Fig. 5). The Direction blocks were randomly presented within each Gesture condition. Analyses have been run on the 6 Direction blocks.

Participants were seated on a stool in front of a desk provided with a support for the eye tracking system allowing pupil recording. Once the sensors for the autonomic recording were installed, participants head was blocked with a chin-and-front-rest. During the experiment, participants were asked to place their hands in a comfortable position on the table, to avoid movements and to face the 17-inch screen (with a 1024*768 resolution) located 60 cm from the head support.

The experiment was run using PsychoPy v1.90.2 and started with the instructions on the screen (“Des vidéos d’une personne réalisant des gestes vont apparaitre dans l’écran. Regardez simplement chaque geste. Appuyez sur la barre d’espace pour démarrer l’expérience »/ « Videos of a person performing gestures will appear on the screen. Just look at each gesture. Press the spacebar to start the experiment »). Then, another screen appeared inviting participants to close their eyes and relax (“Fermez les yeux et détendez-vous”/“Close your eyes and relax”). This condition lasting 3 min allowed us to record the participants’ resting state and was used as baseline. After the 3 min, an acoustic signal lasting 0.5 s invited participants to open their eyes. On the screen participants were informed that the task was up to start (“La tache va commencer”/ “The task is ready to start”). The “trial block” was presented always as first, then, the Gesture conditions were randomly presented across participants. The gesture Direction blocks in each Gesture condition were also presented in a random order.

Once the task was finished, participants were invited to close again their eyes (“L’expérience est terminée, fermez les yeux et détendez-vous »/ “The experiment is over, close your eyes and relax”). This second resting condition ended with an acoustic signal (0.5 s) that determined the end of the experiment (Fig. 5). The questionnaires were then administered.

### Psychophysiological data recording

The recording of pupillometry was carried out with an SR Research Eyelink 1000 eye-tracker in a desktop mount configuration that was posited below the screen and used to record participants’ pupil dilation at a sampling frequency of 500 Hz. The period of interest was registered between 2 and 5 sec after stimulus onset. Pupil dilation is a continuous variable expressed over time. Since the aim was to evaluate the fluctuations of pupil dilation according to the nature of the stimulus^22^, the mean pupil area dilatation (eyelink arbitrary unit) with respect to baseline (onset of the stimulus) for each video was extracted.

The cardiac activity was recorded at a sampling frequency of 1000 Hz with a BIOPAC Data Acquisition unit amplifier (MP35; BIOPAC Systems, Inc., Goleta, CA, USA) and AcqKnowledge BIOPAC acquisition software installed on a separate laptop.

The ECG signal was band-pass filtered at 0.5 and 35 Hz with a notch filter set at 50 Hz in BIOPAC acquisition software. BIOPAC software was used to calculate the R-R intervals subsequently corrected with a visual examination by an experimenter blind of the experimental hypothesis. For HRV quantification, we referred to the Kubios software user’s guide^48^. To remove the very low frequency components, the R-R intervals were first detrended with a smoothness-prior method (<0.04 Hz^49^). Then, for the baseline resting phase (duration 180 s) and for each Gesture type block lasting 130 s (Meaningless, Pantomime and Intransitive toward and away from the body) a power spectral density analysis was run using a non-parametric method (Fast-Fourier Transform), with a high frequency band set at 0.15–0.4 Hz. The power spectral density was then integrated over the high frequency band and transformed in natural-log. The phasic HF-HRV indexes were derived for each condition by subtracting the HF-HRV expressed in log(ms^2^) registered in each Gesture Direction block to the HF-HRV expressed in log(ms^2^) registered in the baseline condition: ΔHF-HRV(Gesture Direction Block) = HF-HRV(Gesture Direction Block)–HF-HRV(baseline).

### Data analyses

All the analyses were carried out with Statistica software v.13, the significance level was set at 0.05. Normal distribution of data was checked with Shapiro-Wilk’s test and assumption of sphericity by means of Mauchley’s test. Data analyses were run using 3 (Gesture Condition) × 2 (Gesture Direction) ANOVAs for repeated measures and Fisher’s test was used for post-hoc analyses.

Considering that the gestures selected did not differ in terms of accuracy and time of recognition, the physiological results obtained in our experimental task could not be ascribed to a difference among the three gesture types or to where they are directed. Also, since participants have to passively observe gestures and no specific task is required, the physiological responses collected during the experimental task should reflect the physiological responses that the three gesture types (Meaningless, Intransitive and Pantomime) and their directions (executed toward and away from the body) provoke when someone observes them.

## Supplementary information

Supplementary Information

## Data Availability

The data that support the findings of this study are available from the corresponding author (Angela Bartolo) upon reasonable request.
