# Supplementary material for: Gestures convey different physiological responses when performed toward and away from the body
Source: Sci Rep. 2019 Sep 6;9:12862. doi: 10.1038/s41598-019-49318-3 (PMC6731307; doi:10.1038/s41598-019-49318-3)
Supplement: Supplementary file 1 — Supplementary Information [file 41598_2019_49318_MOESM1_ESM.pdf]

Title Page: Appendix

Title of the manuscript: Gestures convey different physiological responses when performed toward and away from the body

By Angela Bartolo, Caroline Claisse, Fabrizia Gallo, Laurent Ott, Adriana Sampaio and Jean-Louis Nandrino

List of gestures:

Meaningless

Toward the body: Hand opened in vertical position, thumb close to the ear, movement toward the face keeping the thumb close to the ear; hand stretched horizontally along the ear opening and closing; back of the hand on the ear opening and closing; hand open on the head, opening and closing; palm of the hand closing the ear on the opposite site of the body, and turning the hand on the back.

Away from the body: index, middle and ring finger in front of the body, horizontally, moving back and forth; thumb and ring fingers linked and the hand moves up and down; index and middle fingers opening and closing in front of the body (back of the hand visible); index and middle fingers moving in front of the body (palm of the hand visible).

Intransitive

Toward the body: gesture of crazy; sign of the cross; perplexity (scratching head); disgusting (gesture of vomiting); being hungry.

Away from the body: gesture of money; waving goodbye; stand up; coming here; go away.

Pantomime

Toward the body: gesture of smoking; combing; putting perfume; lipstick; eating a soup;

Away from the body: gesture of pouring water; putting the salt; using a key; cleaning a blackboard; turning a soup with a ladle.
